# Utility of risky cannabis use concept and the role of standard units for achieving an operational definition

**DOI:** 10.1192/j.eurpsy.2024.121

**Published:** 2024-08-27

**Authors:** H. Lopez-Pelayo, C. Oliveras

**Affiliations:** Hospital Clínic Barcelona, Barcelona, Spain

## Abstract

**Abstract:**

Over the past decade (2010-2019), the number of people admitting to using cannabis in the European Union (including the United Kingdom, Norway, and Turkey) increased by 27%, from 3.1% to 3.9%. Notably, Portugal, Spain, and Luxembourg topped the list with the highest percentages of daily cannabis users among those who had consumed the substance in the last month.

With the relaxation of recreational cannabis laws in various European countries, such as Germany, Malta, and Luxembourg, there is a growing need for a public health-oriented and preventative approach. Drawing parallels with alcohol-related strategies, this session aims to explore this evolving landscape from a clinical perspective.

The focus will be on the World Health Organization’s definition of risky substance use, aiming to make it practical and applicable. Two existing proposals from Canada and Spain will be reviewed, with an emphasis on the role of standardized cannabis units in defining risk and the quest for consensus in this regard.

Additionally, the session will examine the similarities between alcohol and cannabis consumption, looking at the effectiveness of the Standard Drink Unit in early intervention and prevention of alcohol-related problems. Insights from the alcohol domain will be discussed, offering valuable lessons for preventing cannabis-related harm.

**Disclosure of Interest:**

None Declared

